# Eating disorder examination-questionnaire – 7: Construct validity in a sample of portuguese overweight women

**DOI:** 10.1192/j.eurpsy.2021.964

**Published:** 2021-08-13

**Authors:** A.T. Pereira, C. Peixoto, H. Martins, C. Marques, F. Carvalho, A. Macedo

**Affiliations:** 1 Institute Of Psychological Medicine, Faculty Of Medicine, University of Coimbra, coimbra, Portugal; 2 Faculty Of Medicine, University of Coimbra, Coimbra, Portugal; 3 Institute Of Psychological Medicine, Faculty Of Medicine, University of Coimbra, Coimbra, Portugal

**Keywords:** eating disorders, overweight, confirmatory factor analysis, EDEQ7

## Abstract

**Introduction:**

Although the Eating Disorder Examination Questionnaire (EDEQ; Fairburn et al. 2008) is the most used instrument worldwide for the assessment of eating disorders symptoms, its factorial structure considerably varies, which limits its construct validity. Using exploratory factor analisys in data from a sample of overweight women, our group found a three-factors structure of the EDEQ Portuguese version (Peixoto et al. 2013), Although it was in accordance with other psychometric studies (eg. Peterson et al 2007), it was different from the original matrix. Further investigation regarding its factor structure has been conducted, with studies supporting a modified seven-item-three-factors structure (dietary restraint, shape/weight overvaluation, body dissatisfaction) with improved psychometric properties (Grilo et al. 2013, 2015), including with Portuguese samples (Machado et al. 2018; Santos et al. 2019).

**Objectives:**

To analyze if the EDEQ version composed of seven items and three factors is replicated in a Portuguese sample of overweight women.

**Methods:**

The EDEQ was administered to an outpatient sample of 276 women (Mean age= 43.85±11.89 years; Mean BMI=32.82±5.43 Kg/height^2^) attending a weight loss treatment consultation in a public hospital.

**Results:**

Confirmatory factor analysis (CFA) revealed an adequate fit of the EDEQ-7 second order model with three dimensions (χ^2^/df=1.5497; RMSEA=.0452, CFI=.9955, TLI=.9914, GFI=.xxx; p<.001). The EDEQ7 Cronbach’s alphas for the total and its dimensions were α<.70.

**Conclusions:**

Given its good psychometric properties, the overlap of the measurement model with those found with different samples and the reduced number of items, the EDEQ7 will be very useful both in research and clinical settings with/for overweight women.

